# Building an Opt-Out Model for Service-Level Consent in the Context of New Data Regulations

**DOI:** 10.1093/phe/phab030

**Published:** 2022-01-11

**Authors:** A R Howarth, C S Estcourt, R E Ashcroft, J A Cassell

**Affiliations:** UCL Institute for Global Health, UK; Glasgow Caledonian University, UK; City University, UK; Brighton and Sussex Medical School, UK

## Abstract

The General Data Protection Regulation (GDPR) was introduced in 2018 to harmonize data privacy and security laws across the European Union (EU). It applies to any organization collecting personal data in the EU. To date, service-level consent has been used as a proportionate approach for clinical trials, which implement low-risk, routine, service-wide interventions for which individual consent is considered inappropriate. In the context of public health research, GDPR now requires that individuals have the option to choose whether their data may be used for research, which presents a challenge when consent has been given by the clinical service and not by individual service users. We report here on development of a pragmatic opt-out solution to this consent paradox in the context of a partner notification intervention trial in sexual health clinics in the UK. Our approach supports the individual’s right to withhold their data from trial analysis while routinely offering the same care to all patients.

## Background

The General Data Protection Regulation (GDPR) came into force on 25 May 2018 as the European Union’s (EU) updated data privacy and security law (GDPR.EU, 2020). It governs how personal data must be collected, processed and erased within the EU. It supports the need to disclose when data collection occurs and the ‘the right to be forgotten’ which gives individuals the right to ask for their personal data to be deleted. Under GDPR, the data controller determines the purpose and means of personal data processing and must ensure that personal data are safeguarded and processed for clearly defined purposes (Information Commissioner's Office, 2020).

This presents a challenge to trials of low-risk clinic-wide interventions, where ethical oversight committees have long accepted that individual consent is not always required (Weijer *et al.*, 2011; Campbell *et al.*, 2017). For such trials, where all patients are routinely offered the same care, service-level consent can be sought from the lead clinician at each participating clinic. We describe here our pragmatic development of an ethical framework for low-risk clinic-wide interventions, which incorporates the individual’s rights under GDPR.

## Our Study

Our model was developed in the context of LUSTRUM programme of research (https://www.lustrum.org.uk/; RP-PG-0614-20009). The programme included a cluster-randomized crossover trial to compare the effectiveness of a novel method of partner notification for heterosexual people with chlamydia with a standard partner notification approach (Estcourt *et al.*, 2020).

In sexual health, partner notification (also known as ‘contact tracing’) is the process whereby individuals who have been diagnosed with a sexually transmitted infection (STI), notify their sex partners about their infection, enabling the partners to get tested and treated. The trial examined the effectiveness of ‘Accelerated Partner Therapy (APT)’, a new evidence-based approach which aims to speed up this process and which has already been shown to be feasible and acceptable for patients and their sex partners (Estcourt *et al.*, 2012, 2015). In the trial, patients in the intervention arm were given the option to deliver a testing and treatment pack directly (or have the pack posted) to their partners, following a telephone medical assessment of the partner by a healthcare professional. This circumvented the need for sexual partners to attend the sexual health clinic in person. The APT option was offered across the sexual health service during the intervention phase of the trial. During this phase, healthcare professionals routinely offered APT to eligible patients for whom it was clinically appropriate, alongside standard partner notification (whereby patients were encouraged to tell partners about their diagnosed STI and the need for their partners to be tested and treated). During the control phase, only standard partner notification was offered.

## Rationale and Journey to Consent Model

In order to ensure that the ethical framework for our trial of APT was appropriate and proportionate, we considered and took advice on the ethical implications of service-wide consent. The design of the trial would require changed processes in existing routine services (Cassell and Young, 2002), carrying minimal or no risk to the individuals using the service and applying to all eligible patients. It had similarities to the many interventions and refinements which take place in healthcare settings, with or without evaluation. Changes to processes, such as introducing nurse-led services or using an updated laboratory test, are routinely implemented on a rolling basis as new delivery models and interventions are incorporated. These do not require individual consent. We were furthermore concerned that individual consent may contribute to low levels of recruitment numbers, as found in our previous work (Estcourt *et al.*, 2015), and that this might compromise the validity and interpretation of the research (Eldridge *et al.*, 2005; Dal-Ré *et al.*, 2019).

We took advice and discussed plans for consent models and data use with colleagues who had biomedical ethics expertise and with members of our Patient and Public Involvement (PPI) Group, made up of people with a range of different experiences in sexual healthcare and different demographic characteristics. They consistently categorized APT as a complex, low-risk healthcare delivery intervention (Weijer *et al.*, 2011) that could be offered in addition to standard partner notification, as a supplement to existing care. Patients (and their selected sex partners) would have full autonomy whether to take up the offer of APT or not during the intervention period in clinic.

Both the experts and the PPI Group supported the use of service-level consent. We consulted the Medical Research Council’s (2002) applicable guidance and in the light of this, considered that a cluster-randomized design was appropriate and individual consent was required only for the method of partner notification rather than randomization. Assessment of capacity to consent to APT would take place according to standard clinic procedures for assessment of capacity for any individual seeking care within the service. Dal-Ré *et al.* (2019) argue that obtaining written informed consent to participate in a pragmatic trial may disrupt the patient–participant encounter and our PPI Group similarly felt strongly that seeking individual consent was not in the interests of participants as it would ‘get in the way’ of the appropriate and sensitive delivery of a partner notification discussion.

As a result of these discussions, we concluded that the ethical approach for this pragmatic cluster-randomized trial was to seek service-level consent for trial participation from the lead clinician at each participating clinic, rather than individual informed consent from patients. This approach was included in our submission to the UK’s Integrated Research Application System (https://www.myresearchproject.org.uk/) on 13 April 2018, 6 weeks before GDPR came into force.

The Chief Investigator attended the Research Ethics Meeting on 14 May 2018. The Committee was unable to give an ethical opinion on the basis of the information and documentation provided and requested further information, including the following:

Consider and then provide clarification of a process to ensure all patients have knowledge that their data could be used for this study and have agreed to it

We researched current practice and sought expert advice in order to develop a process to meet this requirement. We consulted a range of experts, including members of our Programme Steering Committee. We sought a precedent for adopting an opt-out process but found that none of the prominent researchers in sexual health that we approached about current practice had such a process in place.

The process that we proposed to the Research Ethics Committee followed the most up-to-date guidance on data sharing for health improvement and research within the NHS from NHS Digital (https://digital.nhs.uk/services/national-data-opt-out-programme/supporting-patients-information-and-resources), the body leading nationally on changes in relation to GDPR. This involved providing information on the steps a patient should take to opt out through informative posters displayed in clinic waiting rooms and freely available leaflets in clinic waiting areas.

**Figure 1.**
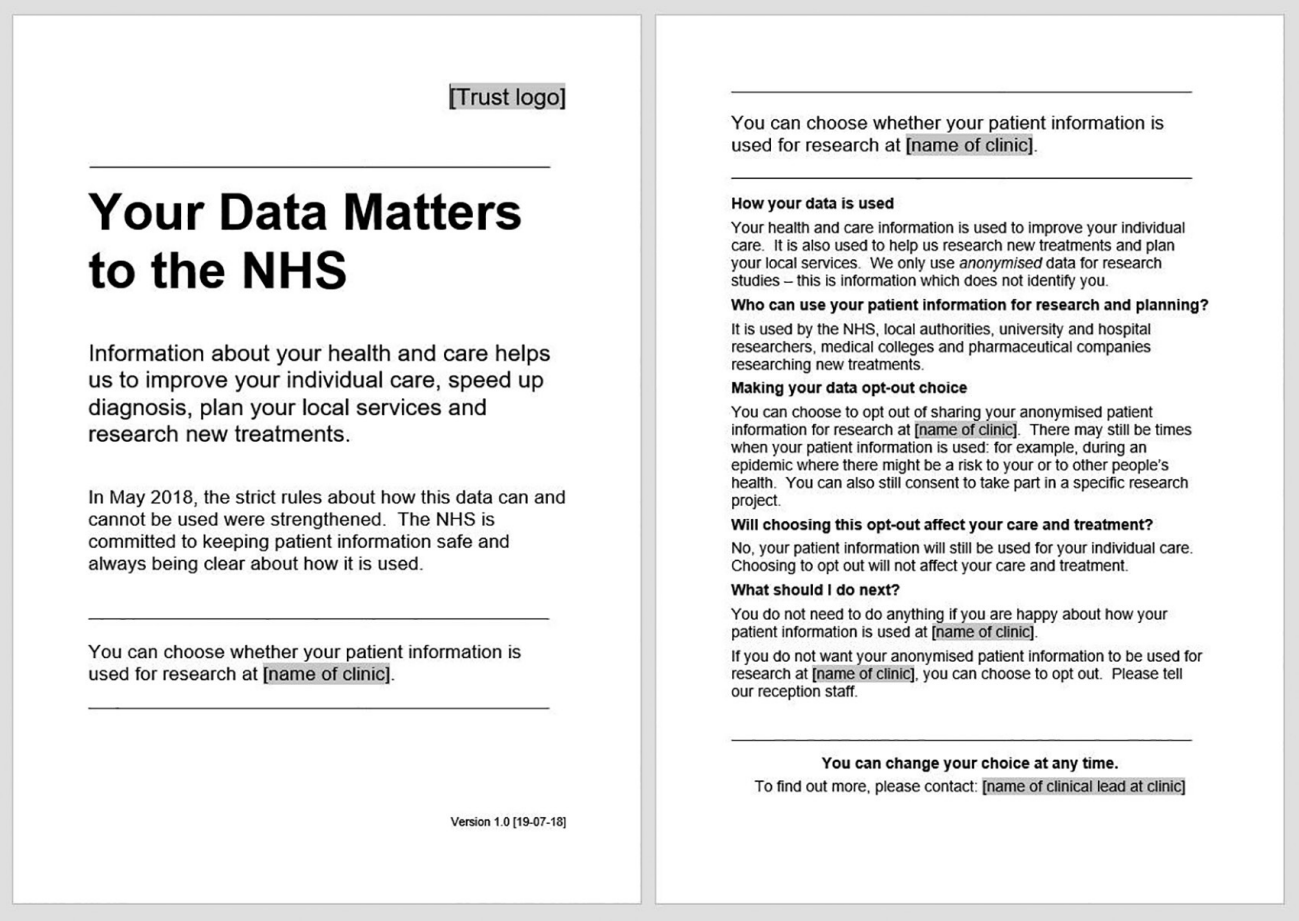
Patient information contained in poster (p1) and leaflet (p1 & 2).

We made minor amendments to refer to the use of anonymized patient information for research within a specific service (Figure 1). In this way, all patients would be informed that their data could be used in the study and were given the opportunity to opt out. We consulted our PPI Group who agreed: ‘This now feels the optimum proposal for use in the proposed research’.

**Figure 2.**
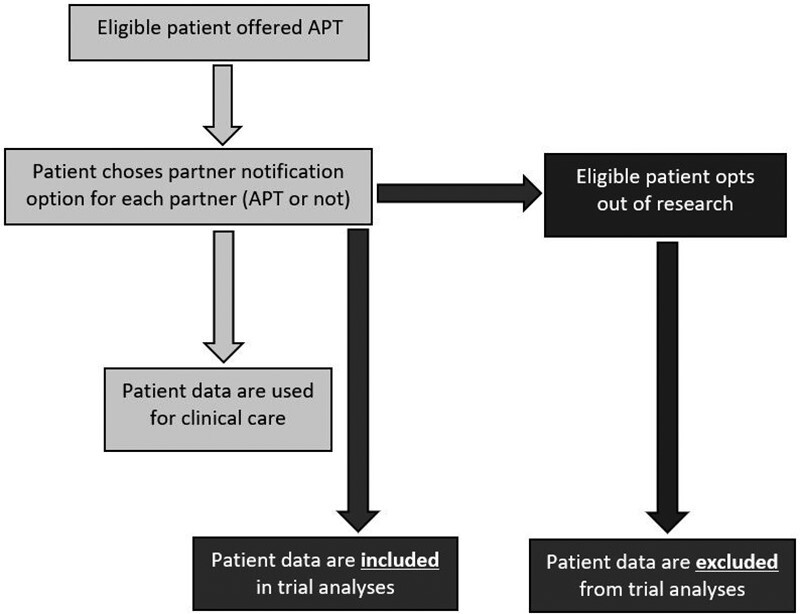
Patient flow for clinical and trial purposes.

Our proposal was accepted by the Chelsea Research Ethics Committee on 23 July 2018 (Ref: 18/LO/0773) and the following procedures were adopted in all participating clinics (Figure 2):

1. During the trial, all partner notification patient data were collected and held securely on RELAY, a bespoke, web-based platform compliant with NHS data storage requirements.
2. The LUSTRUM team provided each clinic with locally adapted copies of the leaflet and poster to be displayed in prominent places around the clinic over the course of the trial.
3. In the leaflet, patients wishing to opt out of research were advised to inform the clinic reception and the clinic collated these responses.
4. Clinics were advised that, as a service-level consent intervention, all patients should be offered the same care irrespective of whether they wished to opt out of their data being used for research purposes. Specifically, they should all be offered APT (if eligible) but be removed from all trial analyses.
5. The research team liaised with the local clinic staff in order to ensure that anyone who had opted out would be removed prior to trial analysis using their unique trial identifier.
6. All clinical data were incorporated into every patient’s clinic record as a routine part of trial processes.

## Case Discussion

Service-level consent has long been regarded as an acceptable ethical approach for some trials of low-risk clinic-wide interventions, where individual consent is not supported for practical reasons and according to scientific argument (Eldridge *et al.*, 2005). However, the implementation of GDPR generated a new circumstance where individuals’ right to opt out from research needed to be included without compromising delivery of the intervention across the entire service in a consistent way. Sheehan *et al.* (2019) propose a broad consent approach for large scale population-level research, arguing that the specific nature of data usage should lie with researchers and research governance. A *Modified Zelen* design was used in a cluster-randomized controlled trial evaluating a complex educational sexual health intervention delivered by healthcare staff (McNulty *et al.*, 2014). Participating GP practices were not informed that they were taking part in the trial in order to avoid biases associated with modified behaviour and participation of sites with particular interest in the initiative. Another cluster-randomized controlled trial to evaluate the use of rapid HIV testing for newly registered adults in general practice adopted *implied consent* for trial participation (Leber *et al.*, 2015), whereby consent is determined by acceptance of the treatment offered. In our trial, the patient’s right to refuse APT as their method of partner notification or opt out from using APT at any time was respected, as it is for any other form of treatment. They did not need to give any reasons for their choice, and it did not affect any further treatment they were due to receive. At the same time, patients had the right to opt out of their data being used for research purposes. In this way, we developed an opt-out model for non-consented data which is GDPR compliant and can be operationalized in sexual health research and service evaluations. Of note, no APT-eligible patients opted out.

Our study raises a number of questions: How could we use service-level consent more widely to conduct better research while protecting individual interests? Should individual consent be sought when services introduce and evaluate quality improvements to their service? Are high-level directives, such as GDPR, too ‘black and white’, rather than proposing considerations to be weighed against each other? How can the potential benefits of such research be incorporated into future guidance and laws?
